# Characterization of the Leaf Microbiome from Whole-Genome Sequencing Data of the 3000 Rice Genomes Project

**DOI:** 10.1186/s12284-020-00432-1

**Published:** 2020-10-09

**Authors:** Veronica Roman-Reyna, Dale Pinili, Frances N. Borja, Ian L. Quibod, Simon C. Groen, Nickolai Alexandrov, Ramil Mauleon, Ricardo Oliva

**Affiliations:** 1 Rice Breeding Platform, International Rice Research Institute, DAPO Box 7777, Metro Manila, Philippines; 2 Present Address: Department of Plant Pathology, The Ohio State University, Columbus, OH USA; 3 Department of Biology, Center for Genomics and Systems Biology, New York University, New York, NY 10003 USA

**Keywords:** *Oryza sativa*, Leaf microbiome, Abundance network, GWAS, Functional profile

## Abstract

**Background:**

The crop microbial communities are shaped by interactions between the host, microbes and the environment, however, their relative contribution is beginning to be understood. Here, we explore these interactions in the leaf bacterial community across 3024 rice accessions.

**Findings:**

By using unmapped DNA sequencing reads as microbial reads, we characterized the structure of the rice bacterial microbiome. We identified central bacteria taxa that emerge as microbial “hubs” and may have an influence on the network of host-microbe interactions. We found regions in the rice genome that might control the assembly of these microbial hubs. To our knowledge this is one of the first studies that uses raw data from plant genome sequencing projects to characterize the leaf bacterial communities.

**Conclusion:**

We showed, that the structure of the rice leaf microbiome is modulated by multiple interactions among host, microbes, and environment. Our data provide insight into the factors influencing microbial assemblage in the rice leaf and also opens the door for future initiatives to modulate rice consortia for crop improvement efforts.

**Supplementary information:**

**Supplementary information** accompanies this paper at 10.1186/s12284-020-00432-1.

## Findings

Plant colonization of terrestrial and aquatic habitats ignited the formation of biodiverse systems, termed phytobiomes. In phytobiomes, plants are in constant interaction with microbial communities that adapted to colonize plant tissues, termed microbiomes (Hassani et al. 2018). Microbial communities that live in association with plants carry a great diversity of metabolic capabilities and often influence broad aspects of plant biology. In agricultural environments, the composition of these communities affects overall crop performance by contributing to important plant functions such as nutrient uptake, environmental responses, and host development (Klein et al. 2012; Naylor et al. 2017; Edwards et al. 2018). For instance, rice seeds studies showed the microbiome as a potential source of plant beneficial bacteria and a source of microbes that could be vertically transmitted (Cottyn et al. 2009; Eyre et al. 2019). For roots and paddy soil microbiomes, several studies identified microbial clusters involved in methane metabolism and nitrogen fixation (Butler et al. 2003; Sessitsch et al. 2011; Bao et al. 2014). Reports on rice rhizosphere microbiome demonstrated associations with vegetative and reproductive host stages and as potential source of biocontrol agents (Spence et al. 2014; Edwards et al. 2015, 2018). Most rice microbiome studies use rRNA gene sequencing and a small sample size. This approach introduces a bias towards diversity and abundance of microorganisms. It also limited statistical power to identify the importance of factors that shape host-microbe interactions (Louca et al. 2018). An alternative to rRNA gene sequencing is shotgun sequencing with large-scale databases. This approach provides more information about the composition and structure of microbiome and also increases confidence about correlations among microbes, environment, and host.

As part of the 3000 Rice Genomes Project (3 K-RGP), 3024 rice accessions were sequenced (The 3000 rice genomes project 2014; Wang et al. 2018). The 3 K-RGP panel has been successfully used to identify the rice genetic architecture underlying several complex morphological and phenological traits (Wang et al. 2018). These whole-genome-*shotgun sequenced* reads capture rice reads and also total DNA of resident microbial communities. The use of non-plant reads provides access to an impressive microbiome dataset in which one can systematically probe the role of environment vs. genotype in dictating microbial abundance. Based on that hypothesis, we extracted non-plant sequences from the 3 K-RGP raw sequence data to characterize the rice leaf microbiome (Additional file 1: Figure S1 and Additional file 2). We used bacterial and archaea reads since it constitutes 86% of the total reads.

## Diversity of the Rice Leaf Microbiome

**Fig. 1:**
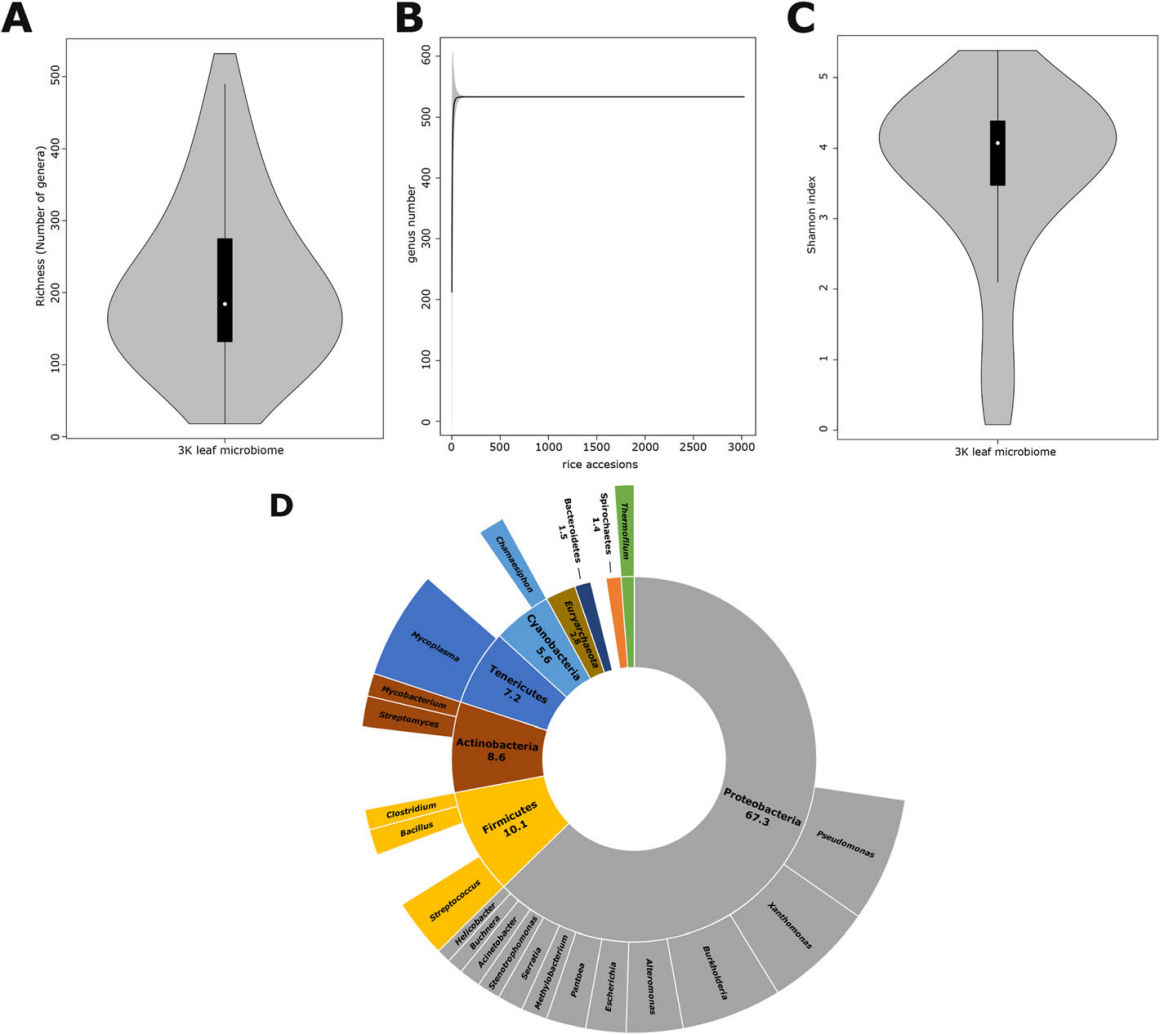
Composition of leaf bacterial community in the 3 K rice genome project. **a**, **b** total leaf bacterial and archaea genera (**a**) and accumulation curves (**b**) in 3024 rice genome accessions. The shade on the curve represents the confidence interval of two in the curve points. **c** Shannon index in all rice accessions. In the violin plots, the black box plot indicates the 75th and 35th percentile. The white dot represents the media. **d** Relative abundance at phylum and genera level. The inner position of the sunburst chart represents phylum and the outer position represents Genus. The chart shows average relative abundance higher than 1% across all samples

We were able to capture and classify Bacterial and Archaea reads from the 3 K-RGP raw database (Additional file 3: Table S1). On average rice leaves of each accession harbor 212 + 111 genera (Fig. 1a). The accumulation curve indicated that unmapped reads captured most of the expected taxa. The accumulation curve reached a plateau around 520 bacteria after the first 100 rice accessions (Fig. 1b). The average Shannon index was 3.64 + 1.2 similar to seed and root rice microbiome (Edwards et al. 2015; Eyre et al. 2019) (Fig. 1c).

We assessed the taxonomic composition at phylum and Genus levels. We found nine Phyla and 23 genera with an abundance higher than 1% (Fig. 1d)*.* The phylum Proteobacteria, common in other leaf microbiomes, was the most abundant group (Rastogi et al. 2012; Bodenhausen et al. 2013; Wallace et al. 2018). Interestingly, the phylum Euryarchaeota, which includes methanogenic Archaea, was marginally present in the aerobic phyllosphere (Knief et al. 2012; Edwards et al. 2015) (Fig. 1d). The aerobic conditions in the phyllosphere could also explain why we did not detect members of taxa commonly found in the soil or rice rhizosphere (Bao et al. 2014; Edwards et al. 2015). The genera *Pseudomonas, Xanthomonas, Mycoplasma,* and *Burkholderia* were the most abundant bacteria in the leaf microbiome (Fig. 1d). *Pseudomonas, Xanthomonas,* and *Burkholderia* have rice pathogenic species. This could indicate that the rice microbiome has a mixture of commensals, beneficial and pathogenic species (Klein et al. 2012). The presence of *Mycoplasma* and other four genera associate with human or animal hosts might be explained by animal and human interaction with the rice cultivation (Additional file 3: Table S2). These interactions should be consider as a way of microbial horizontal acquisition (Sasaki et al. 1999; Cottyn et al. 2009; Campisano et al. 2014).

The 3 K-RGP contained accessions grown in the Philippines (agPh) and in China (agCh). To further dissect the microbial community composition, we assessed taxa richness in each location. We found that the Shannon index for agCh was 4.08 while for agPh the index was 3.39 (Additional file 1: Figure S2). For genus richness, we found that that agCh contains on average 186 genera while agPh contained 152 genera. Then we compared the relative abundance of Bacteria and Archaea at different taxonomical levels. The differences between locations were mainly explained by genera. Forty-eight genera contributed to 70% of the differences between locations; 25 of them had a relative abundance higher than 1% (Additional file 3: Table S3). Other studies have shown that environmental variation appears to be the major driver of microbiome diversity (Peiffer et al. 2013; Okubo et al. 2014; Copeland et al. 2015; Edwards et al. 2015, 2018; Wagner et al. 2016; Moronta-Barrios et al. 2018). Thus, it is likely that the differences between agCh and agPh might be associated with the exposed to an distinct array of microbes.

We were aware that the manipulation of samples during gDNA extraction or sequencing might include bacteria not commonly present in rice. To rule out that abundant genera were not artificially introduced during sample collection, we used qPCR to detect 11 highly abundant genera in 17 rice accessions from our 3 K-RGP panel (Additional file 2 and Additional file 3: Table S4). We used new plants with the idea that the genera we found in 3 K panel are common members of the rice leaf microbiome. We were able to quantify the presence of all taxa and observed a similar distribution across accessions (Additional file 3: Table S4). Similar to our previous findings, the genera *Pseudomonas, Xanthomonas*, and *Mycoplasma* were the most abundant genera, ruling out that highly abundant genera were introduced artificially. Overall, our data showed that environmental variation play a key role in determining variation in microbial community composition (Hartmann et al. 2015).

## The Functional Profile of the Leaf Microbiome

**Fig. 2:**
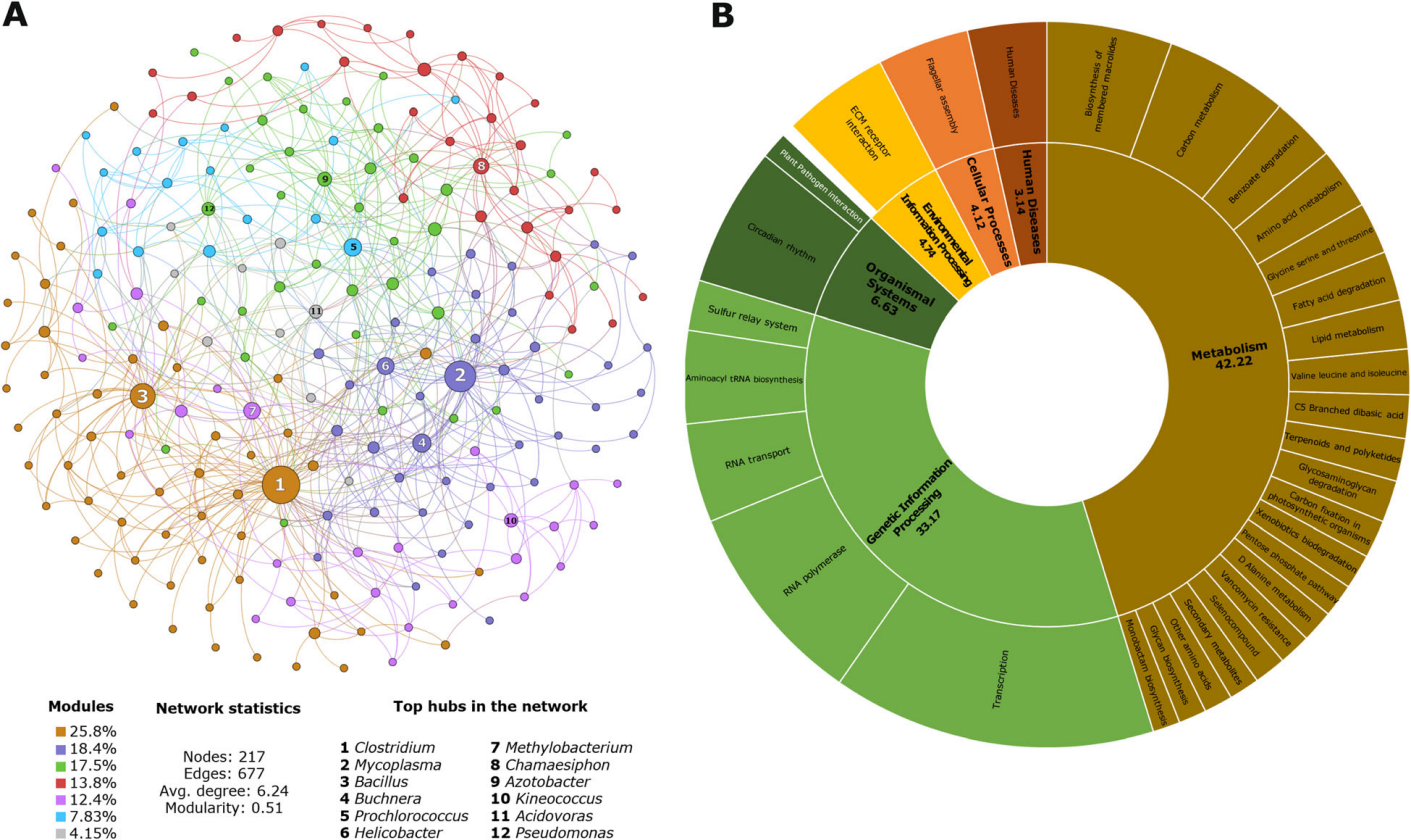
Structure and functional profile of the rice leaf bacterial communities. **a** Microbial ecological network from the 3 K microbiome with abundant genera present in at least 50% of all samples. The colors represent the seven modules in the network. Each node represents a genus and the circle size indicates betweenness centrality increment. For the network analysis the genus counts were center-log-transformed. **b** KEGG pathways entries with more than 1% relative abundance in all rice accessions. The inner circle indicates KEGG class 1 pathways and the external circle indicates classes 2 or 3

Networks of interactions among microbes further shape the establishment and maintenance of the microbial community (Horton et al. 2014; Layeghifard et al. 2017; Hassani et al. 2018). In those networks, highly connected genera or “hubs” play an important ecological role in the establishment of the community and the regulation of microbial assembly (Agler et al. 2016; Layeghifard et al. 2017; Hassani et al. 2018). To determine the structure of the rice leaf microbiome, we inferred microbial co-occurrence networks and identified critical hubs (Additional file 2). The criteria to select the hubs was based on a combination of the network properties such as weighted degree, betweenness centrality, modularity class, clustering, and eigen-centrality (Additional file 3: Table S5). We found that the rice microbe network can be defined by 12 hubs: *Clostridium, Mycoplasma, Bacillus, Buchnera, Prochlorococcus, Helicobacter, Methylobacterium, Chamaesiphon, Azotobacter, Kineococcus, Acidovorax, and Pseudomonas* (Fig. 2a, Additional file 3: Table S5). Interestingly, connectivity of a genus within the network did not correlate with their abundance. For example, the highly abundant genera *Xanthomonas* and *Burkholderia* were not identified as hubs. The genera *Kineococcus* or *Helicobacter*, with less than 1% abundance, played a role in shaping the network of interactions (Agler et al. 2016).

The hubs *Methylobacterium*, *Pseudomonas*, *Bacillus*, *Kineococcus, Azotobacter, Acidovorax*, and *Clostridium* have also been reported as commensal, beneficials, or pathogens in other plants (Cottyn et al. 2009; Bian et al. 2012; Horino et al. 2015; Chen et al. 2018; Lai and Huang 2018). The hubs *Chamaesiphon* and *Prochlorococcus* could be part of the rice microbiome, as the paddy field conditions like shallow water and sunlight could create an ideal environment for algae growth. The hub *Buchnera,* could be part of aphids and rice interaction, as *Buchnera* is an aphid endosymbiont. The hubs *Mycoplasma* and *Helicobacter*, associated with human diseases, have not been associated with plant microbiomes. We hypothesized that these bacteria, generally present in animals and humans, are hubs in the rice microbiome due to a strong influence of agricultural practices, like irrigation water or crop-human interaction.

The networks can be further sub-structured into modules, which represent a group of organisms that has more interactions within the group than with other members of the network (Layeghifard et al. 2017). We found seven modules. Based on the bacterial composition of some modules, microbial genetic ancestry or ecological niche seems to shape them (Fig. 2a, Additional file 3: Table S6). For example, one module was enriched with Cyanobacteria, another module had bacteria associated to animals and other modules mainly had Alphaproteobacteria or Betaproteobacteria associated with plants or soils (Additional file 3: Table S6). Overall, taxonomy status or ecological niche rather than the abundance of individual taxa appears to define the interactions within the microbial community. Moreover the number of modules in the network suggested a highly stable network since a microbial community appears to reach an equilibrium when its network of interactions had a small number of modules (Layeghifard et al. 2017).

The functional and nutritional capacities of the microbes partly define the networks of interactions among microbes (Agler et al. 2016; Layeghifard et al. 2017; Hassani et al. 2018). To understand if biological functions in the bacterial communities associate to the ecological network, we predicted functional categories for the microbial taxa (Additional file 2). We found 85 predicted KEGG functional categories, where 28 had more than 1% abundance (Fig. 2b, Additional file 3: Table S7). Transcription, translation, primary metabolism, flagellar assembly, environmental adaptation (ECM receptor interaction) and secondary metabolism (terpenoids, antibiotics, and xenobiotics) were the most abundant pathways. The enrichment of these KEGG pathways linked with the abundant genera as well as the bacterial hubs. For example, the enrichment of pathways related to human diseases reflected the presence of animal pathogens in the rice microbiome. Moreover, *Pseudomonas,* facultative anaerobes, methanogenic bacteria, and some gram-positive bacteria, like *Bacillus* and *Streptomyces*, have the capacity to metabolize xenobiotic, terpenoids and antibiotics compounds (Additional file 3: Table S2). The xenobiotics metabolism KEGG category suggested an adaptation of the bacterial to an environment where chemicals, like pesticides, might be applied to crop fields (Tipayno et al. 2017). The presence of functional categories common to other leaf microbiomes, restated the idea that functions and communities are not random (Delmotte et al. 2009; Xiao et al. 2017). Moreover, our results aligned with the idea that metabolic functions and interaction within the microbiome regulate the establishment of the community (Agler et al. 2016; Louca et al. 2017).

We then determined the microbial co-occurrence networks and functional categories for each agCh and agPh dataset. Interestingly, agCh and agPh assembled networks with similar average degree and modularity (Additional file 1: Figure S3A). We identified seven highly connected hubs in each dataset, five of which were common to agCh and agPh networks (Additional file 1: Figure S3A, Fig. 2). Furthermore, we found no significant differences between agCh and AgPh functional profiles, where both datasets shared 22 of 24 KEGG level pathways (Additional file 1: Figure S3B). The data suggest that rice leaf microbiome assemble communities with similar structure, independently from the available microbial diversity. Overall, the similarity of the network structures and the functional redundancy of the leaf microbiome dataset supported the idea that key microbial groups might regulate the establishment by providing essential functions in the community (Hassani et al. 2018). This result also aligned with other studies where core biological functions of the microbiome are associated to different plant tissues or plant substrates (Vorholt 2012; Agler et al. 2016; Louca et al. 2017; Xiao et al. 2017).

## Rice Genetic Associations with the Leaf Microbiome Composition

The interactions of environment, microbes, and hosts modulate the assembly of the microbial community (Layeghifard et al. 2017; Hassani et al. 2018; Wallace et al. 2018). The host might play a key role, as genetic control on the microbiome assembly has been reported in Arabidopsis and *Nicotiana benthamiana* (Long et al. 2010; Lebeis et al. 2015). To identify rice genetic factors that control the recruitment and establishment of specific microbial players, we conducted a genome-wide association study (GWAS) on 3024 rice accessions, using the genomic information from 6.5 million SNPs and the relative abundance of the 12 hubs (Additional file 2). To avoid association bias due to lack of information for most of the Chinese accessions, we kept agCh and agPh together for the analysis.

**Fig. 3:**
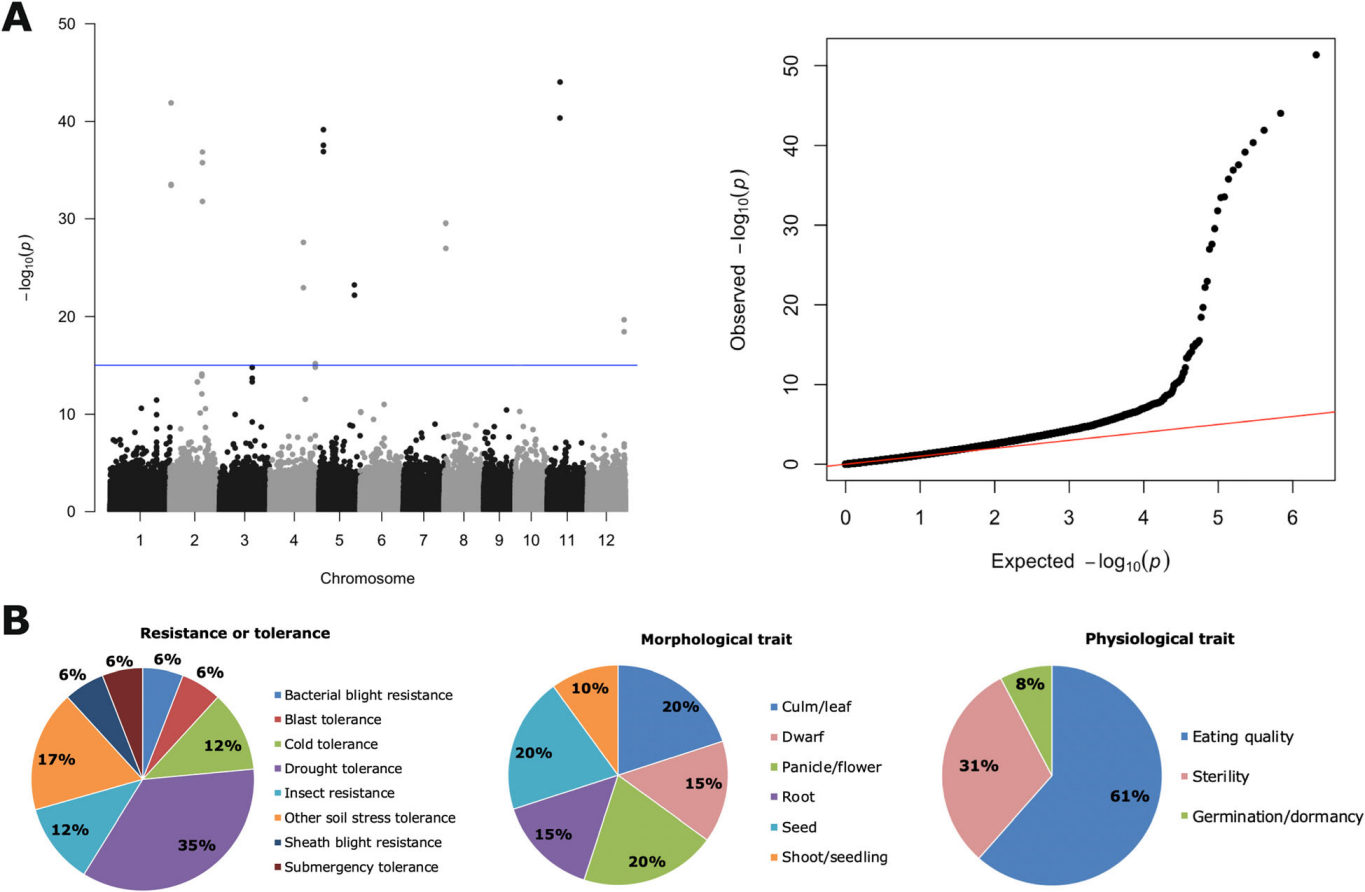
Rice metabolic pathways are associated with the microbiome structure. **a** Genome-wide association for the 12 microbial hubs simultaneously using a multivariate linear mixed model with GEMMA software. Left panel, Manhattan plot, using the rice dataset, indicated the major peaks (significant SNPs) distributed across six chromosomes, associated with microbial abundance of the 12 hubs. Right panel, Quartile–quartile plot for expected versus observed −log(P) values. *P*-values were adjusted with FDR and values lower than 1E-15 were consider significant (blue line). **b** the significant SNPs found in this study co-localized with a number of agronomic QTLs retrieved from Q-TARO database, associated with resistance or tolerance; morphological trait; and physiological trait

Overall, we found 22 significant SNPs shared among the 12 hubs abundance (*P*-value <1E-16, ci = 0.95), distributed across six chromosomes (Fig. 3a). Twenty of the 22 SNPs located within 11 annotated rice genes (Additional file 3: Table S7). Seven genes had at least one SNPs with a missense effect. The seven genes were catalase isozyme A, malate synthase, inorganic H+ pyrophosphatase, endo-1,4-beta-xylanase, similar to ClpC, similar to ATP-dependent Clp protease, and 5-methyltetrahydropteroyltriglutamate--homocysteine methyltransferase. Most of the seven genes had annotations for stress responses, carbon metabolism and regulation of gene expression (Additional file 3: Table S8). We then determined the relationships between significant SNPs and hubs abundance (Additional file 2). The average of the 12 hub abundances (centered log-ratio normalized) showed significant differences in four haplotypes, Chr4–32,927,447, Chr5–14,856,070, Chr5–14,856,078, and Chr5–25,806,324 (Additional file 1: Figure S4A). The SNPs related to chromosome five were the genes endo-1,4-beta-xylanase and a heat shock protein (Additional file 3: Table S8). We also looked at the relationship between significant SNPs and the hubs with higher betweenness centrality, *Mycoplasma, Clostridium* and *Bacillus*. The three bacteria had significant differences for the SNP Chr4–32,927,447. *Mycoplasma* had significant differences with 18 more haplotypes. *Clostridium* with one more and *Bacillus* two more. Based on the 22 significant SNPs and the differences found with hub abundances, it seems the regulation of the microbial community could be associated with less toxic or carbon-enriched host environments.

From the 22 SNPs, we identified 12 haplotype blocks spanning 120 candidate genes using linkage disequilibrium analysis (Additional file 2 and Additional file 3: Table S8). Gene ontology (GO) enrichment analysis, with the 120 genes, indicated physiological process, intracellular, membrane-bound organelle and catalytic activity as the most abundant (> 50%) GO terms (Additional file 1: Figure S5 and Additional file 2) (Liu et al. 2013). To explore if the 120 gene were enriched and previously described to be associated with rice agronomic traits, we used the rice quantitative trait locus (QTL) database, Q-TARO. Overall, the 120 candidate genes mapped to 42 QTLs distributed in different categories: resistance or tolerance (15 QTLs), morphological traits (16 QTLs), and physiological traits (11 QTLs) (Fig. 3b, Additional file 3: Table S9). Drought tolerance was the most abundant trait in resistance or tolerance QTL. The presence of drought QTL followed by other traits related to abiotic stimuli validated the idea that host responses to the environment can be associated with the microbiome composition (Long et al. 2010; Lebeis et al. 2015; Naylor et al. 2017). The morphological traits Culm/leaf, Panicle/flower, and seed traits were equally abundant. The traits associated with different plant tissues validate the idea of a dynamic microbiome that shifts in different host tissue or developmental conditions, like vegetative or reproductive (Edwards et al. 2018). The seeds trait, the eating quality as physiological trait, and the QTL associated with grain chalkiness, suggested that seeds quality are a key factor in the vertical transmission and shaping of the microbiome (Cottyn et al. 2009; Eyre et al. 2019). Similar to other studies, we found that host responses to stress, primary pathways, and plant tissue morphology, are perhaps, common host genetic factors related to leaf microbiome assembly (Horton et al. 2014; Wallace et al. 2018). In that scenario, is likely that allelic variation of certain rice genes influences the composition of plant microbiome or of particular groups.

## Conclusions

Our study explored the idea that information about the composition of leaf microbial communities of rice plants can be extracted from the raw host genome sequence data. Using the 3 K-RGP dataset, we were able to describe the composition and structure of the rice leaf microbiome. We validated the idea that microbiomes do not assemble randomly but that their formation is governed by complex interactions among microbes, host, and environment. Given the scale of the 3 K-RGP dataset, we took the first steps in unearthing the factors behind rice leaf microbiome assembly by using GWAS and microbiome abundance as a trait. The next steps will be to understand how the microbiome from roots, soil, seeds, and leaves transmit among tissues and the interaction with fungal and virus microbiome. This study leaves open questions on the benefit of these hubs but also on the host mechanisms that can be used to modulate the community for crop improvement purposes.

## Supplementary information

**Additional file 1: Figure S1.** Generation of 3000 rice genomes dataset and pipeline for collecting the leaf microbiome. **Figure S2.** Growing location shapes the rice leaf microbiome diversity and composition. A-B Richness and Shannon index comparisons between accessions grown in China and Philippines; *P-value < 0.001. Kruskal-Wallis test. C Leaf microbiome composition of rice accessions grown in China and Philippines. The inner position of the sunburst chart represents taxonomic hierarchy phylum and the outer position represents Genus. The chart shows abundance higher than 1% determined as the relative abundance across all samples. The black line highlights the unique genera for each environment. The figure showed the average relative abundance across all accessions from each location using only the classified reads. **Figure S3.** Leaf microbiome network and functional profile is conserved across growing locations. A Microbial ecological network from China and the Philippines with abundant genera present in at least 50% of all samples. The colors represent the seven modules of each network. Each node represents a genus and the circle size indicates betweenness centrality increment. The key microbial hubs are *Clostridium (Clo), Mycoplasma (My) and Helicobacter (H).* Other hubs in China are *Spiroplasma (Sa), Azospirillum (Am), Prochlorococcus (Pr), Sphingobium (Sm).* For the Philippines, important hubs are *Bacillus (Ba), Pseudomonas (P),* and *Azotobacter* (A). The properties of the network are number of edges, number of nodes or genera, average degree and modularity. Only for the network analysis the genus counts were center-log-transformed. B KEGG level 2 pathways with more than 1% relative abundance in accessions grown in China and the Philippines. NS no significant, Wilcoxon rank-sum test = 6869, *P*-value = 0.421. **Figure S4.** Relationships between significant SNPs and hubs abundances represented as box plots. A significant difference using the average of the 12 hubs abundances. B Significant differences using the hubs *Mycoplasma, Clostridium* and *Bacillus* abundances. All box plots have an FDR adjusted *p*-value < 0.05 using a pairwise Wilcoxon test. SNPs nomenclature are chromosome and position. **Figure S5.** Gene ontology enrichment analysis with the genes from the haploblocks. Bars indicate the frequency of the three GO categories was calculated over the 120 genes. The colors in the bars indicate the -log(p-value) for each GO term.

**Additional file 2.** Methods.

**Additional file 3: Table S1.** List of 3 K-RGP rice accessions with number of reads that did not map to the rice genomes (unmapped reads). **Table S2.** Phyla and genera composition of the 3 K RGP microbiome. **Table S3.** Significant genera that contribute to the differences between accessions grown in China and accessions grown in Philippines. **Table S4.** Quantitative PCR validation of metagenomic analysis using 17 rice accessions from the 3 K-RGP project. The table indicate the genera used for the experiments, followed by the primer sequences, the reference for the primers, the qPCR results (delta Ct and standard deviation). Then the list of the 17 accessions and their groups are indicated. **Table S5.** Co-abundance network values for the most abundant genera in the 3KRGP microbiome. **Table S6.** Co-abundance network modules with the most connected microbes. **Table S7.** Metabolic pathways predicted by Vikodak based on KEGG levels 1 and 3. **Table S8.** Significant signals from the genome wide association analysis (GWAS). **Table S9.** Description of haplotype blocks for each significant SNP, number of associated candidate genes and the QTLs that match to the same region.

## Data Availability

All data generated or analyzed during this study are included within figures, tables and the supplemental material. The 3 K SNPs were downloaded from https://snp-seek.irri.org/.

## Abbreviations

GWAS: Genome-wide association studies
rRNA: Ribosomal ribonucleic acid
3 K-RGP: 3000 Rice Genomes Project
qPCR: Real-time polymerase chain reaction
SNPs: Single-nucleotide polymorphisms
QTL: Quantitative trait locus
KEGG: Kyoto Encyclopedia of Genes and Genomes
FDR: False discovery rate
GO: Gene ontology
