# Supplementary material for: Characterization of the Leaf Microbiome from Whole-Genome Sequencing Data of the 3000 Rice Genomes Project
Source: Rice (N Y). 2020 Oct 9;13:72. doi: 10.1186/s12284-020-00432-1 (PMC7547056; doi:10.1186/s12284-020-00432-1)
Supplement: Supplementary file 1 — Additional file 1: Figure S1. Generation of 3000 rice genomes dataset and pipeline for collecting the leaf microbiome. Figure S2. Growing location shapes the rice leaf microbiome diversity and composition. A-B Richness and Shannon index comparisons between accessions grown in China and Philippines; *P-value < 0.001. Kruskal-Wallis test. C Leaf microbiome composition of rice accessions grown in China and Philippines. The inner position of the sunburst chart represents taxonomic hierarchy phylum and the outer position represents Genus. The chart shows abundance higher than 1% determined as the relative abundance across all samples. The black line highlights the unique genera for each environment. The figure showed the average relative abundance across all accessions from each location using only the classified reads. Figure S3. Leaf microbiome network and functional profile is conserved across growing locations. A Microbial ecological network from China and the Philippines with abundant genera present in at least 50% of all samples. The colors represent the seven modules of each network. Each node represents a genus and the circle size indicates betweenness centrality increment. The key microbial hubs are Clostridium (Clo), Mycoplasma (My) and Helicobacter (H). Other hubs in China are Spiroplasma (Sa), Azospirillum (Am), Prochlorococcus (Pr), Sphingobium (Sm). For the Philippines, important hubs are Bacillus (Ba), Pseudomonas (P), and Azotobacter (A). The properties of the network are number of edges, number of nodes or genera, average degree and modularity. Only for the network analysis the genus counts were center-log-transformed. B KEGG level 2 pathways with more than 1% relative abundance in accessions grown in China and the Philippines. NS no significant, Wilcoxon rank-sum test = 6869, P-value = 0.421. Figure S4. Relationships between significant SNPs and hubs abundances represented as box plots. A significant difference using the average of the 12 hubs abundance [file 12284_2020_432_MOESM1_ESM.pdf]

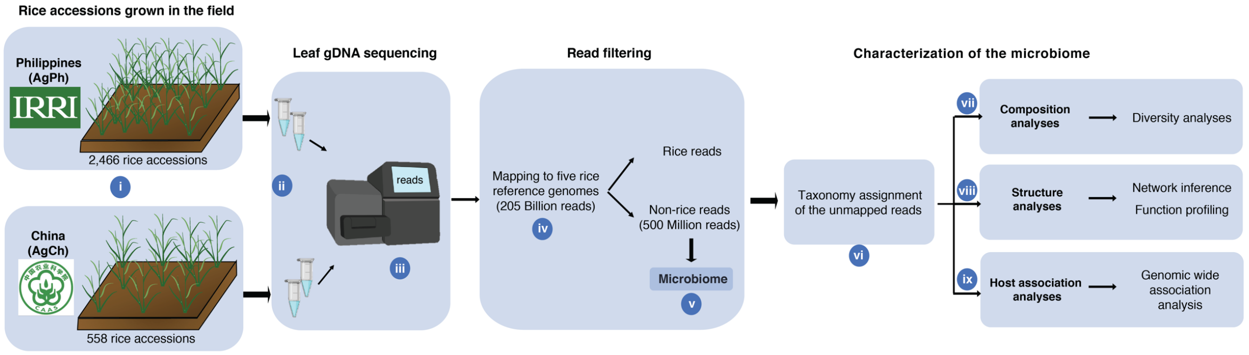

Figure S1. **Generation of 3000 rice genomes dataset and pipeline for collecting the leaf microbiome.** **i)** Selected gene bank accessions seeds were grown in the Philippines (agPh) or China (agCh). The Philippines accessions belong to the International Rice Gene bank Collection (IRGC) at the International Rice Research Institute (IRRI). The accessions grown in China are part of a bigger collection from the China National Crop Gene Bank (CNCGB) in the Institute of Crop Sciences, Chinese Academy of Agricultural Sciences (CAAS). The rice accessions from IRRI were grown in the Philippines field and the accessions from CAAS were grown in China field(The 3,000 rice genomes project 2014). **ii)** Genomic DNA (gDNA) was extracted from leaves of young plants for each sampled accession by modified CTAB method. Each accession consisted of a pool of samples. **iii)** All accession genomes were sent to BGI group (https://www.bgi.com) to construct the libraries and do the sequencing with the HiSeq2000 platform. **iv)** Clean reads, that correspond to 205,084,357,762 pair-end reads for all 3,024 genomes, were mapped to five reference genomes using the BWA software. The reference genomes were Nipponbare, 93-11, IR64, Kasalath, and DJ123(Wang et al. 2018). We separated the reads that map to all rice genomes from the reads that did not map to any rice genome. **v)** We suggested the reads that did not map to any of the rice genomes (non-rice reads) came from microbial DNA that cohabits with rice. The microbes might be present on the rice leaf surface (epiphytes) and inside the leaves (endophytes) microbes. **vi-ix)** We then proceeded with the characterization of the microbiome. **vi)** First, we assigned taxa to the unmapped reads and calculated abundance of microbes. We used an NCBI dataset for Bacteria and Archaea. **vii)** Then, we analyzed the composition of the rice microbial community using diversity. **viii)** We analyzed the structure of community by inferring ecological networks and by predicting function profiles. **ix)** Lastly, we used the single nucleotide polymorphisms database from rice (SNPseek) to look for genomic traits associated with the microbiome abundance.

References

The 3,000 rice genomes project (2014) The 3,000 rice genomes project. GigaScience 3:7. https://doi.org/10.1186/2047-217X-3-7

Wang W, Mauleon R, Hu Z, et al (2018) Genomic variation in 3,010 diverse accessions of Asian cultivated rice. Nature 557:43. https://doi.org/10.1038/s41586-018-0063-9

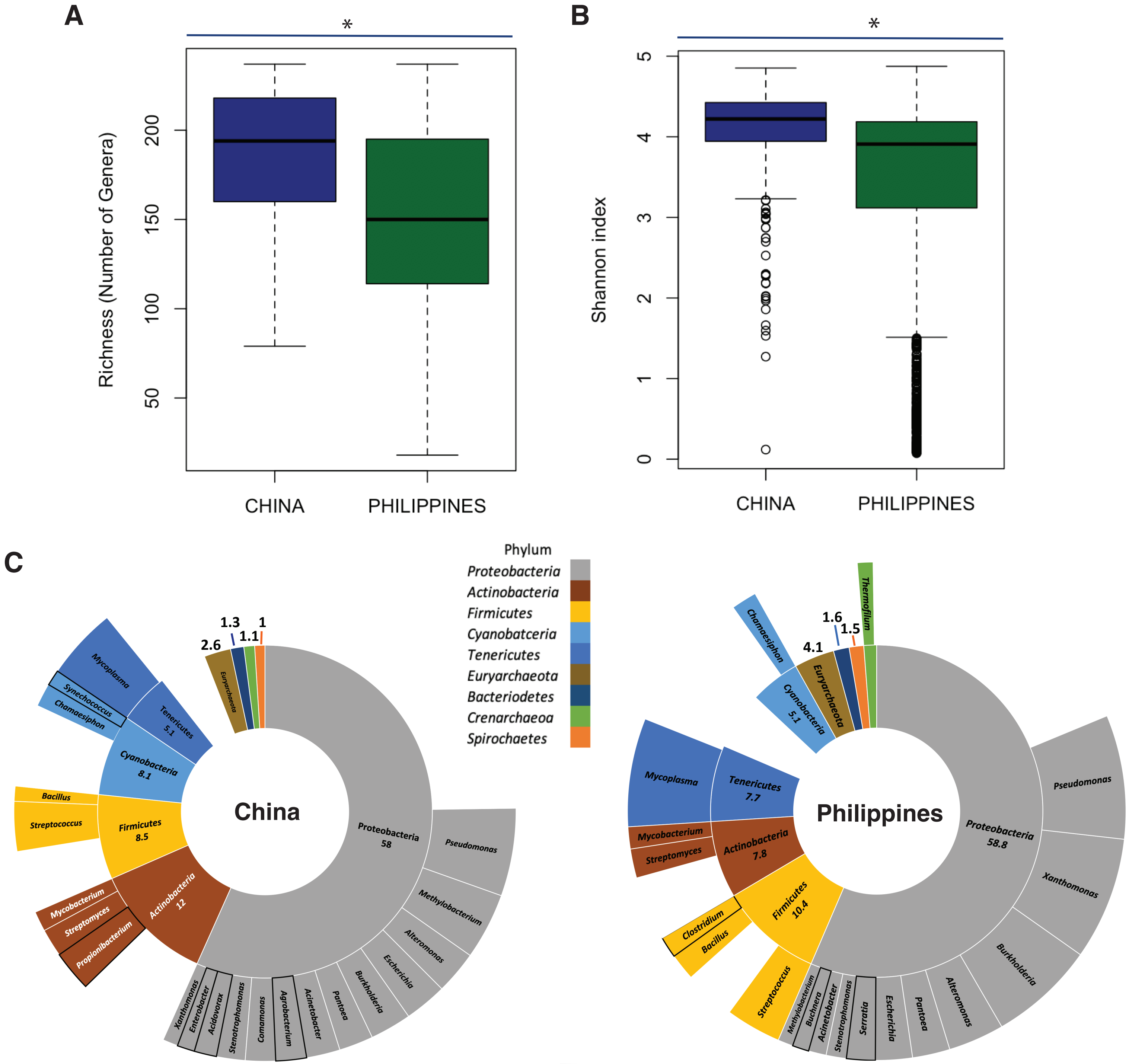

Figure S2: Growing location shapes the rice leaf microbiome diversity and composition.

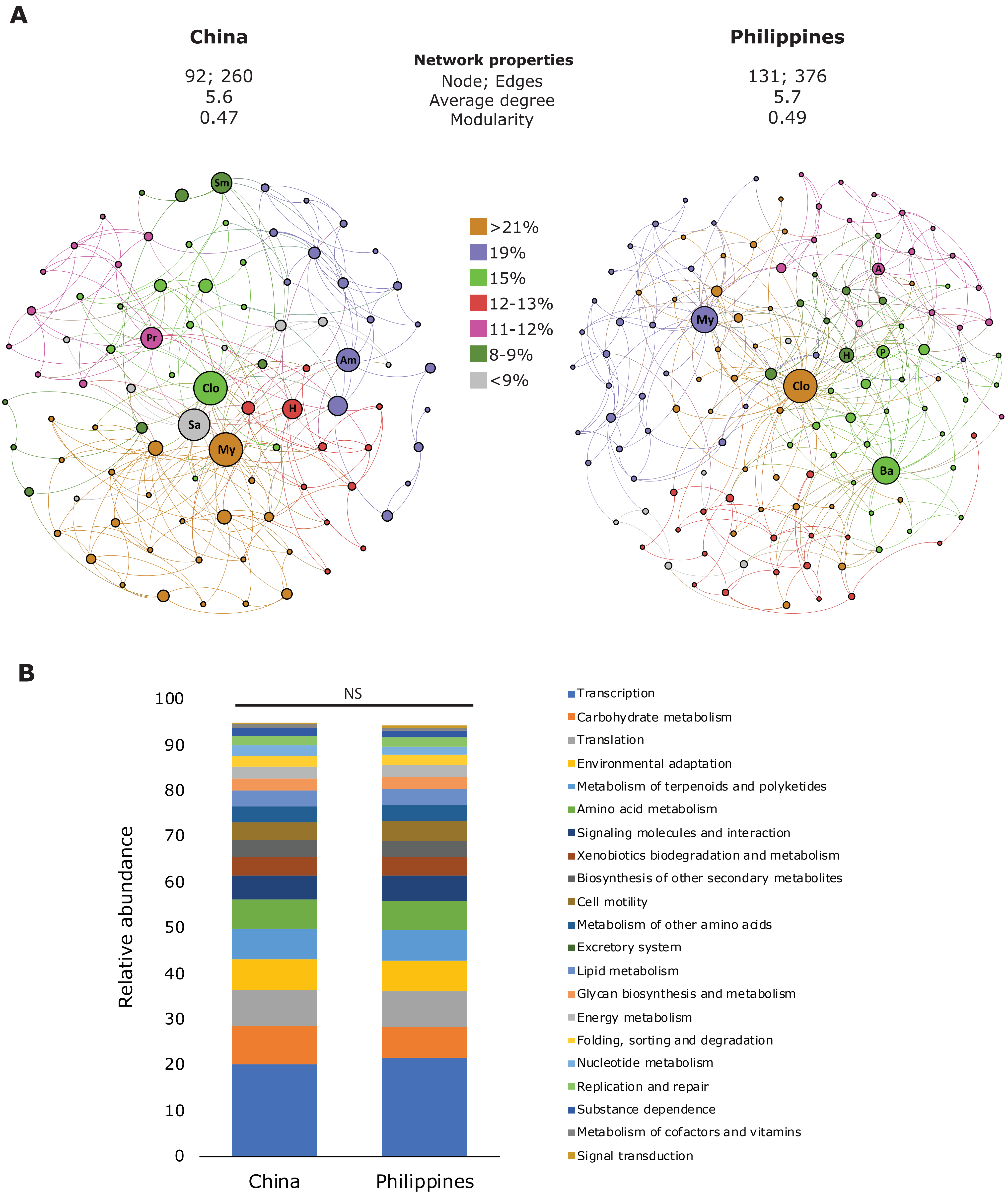

Figure S3: Leaf microbiome network and functional profile is conserved across growing locations.

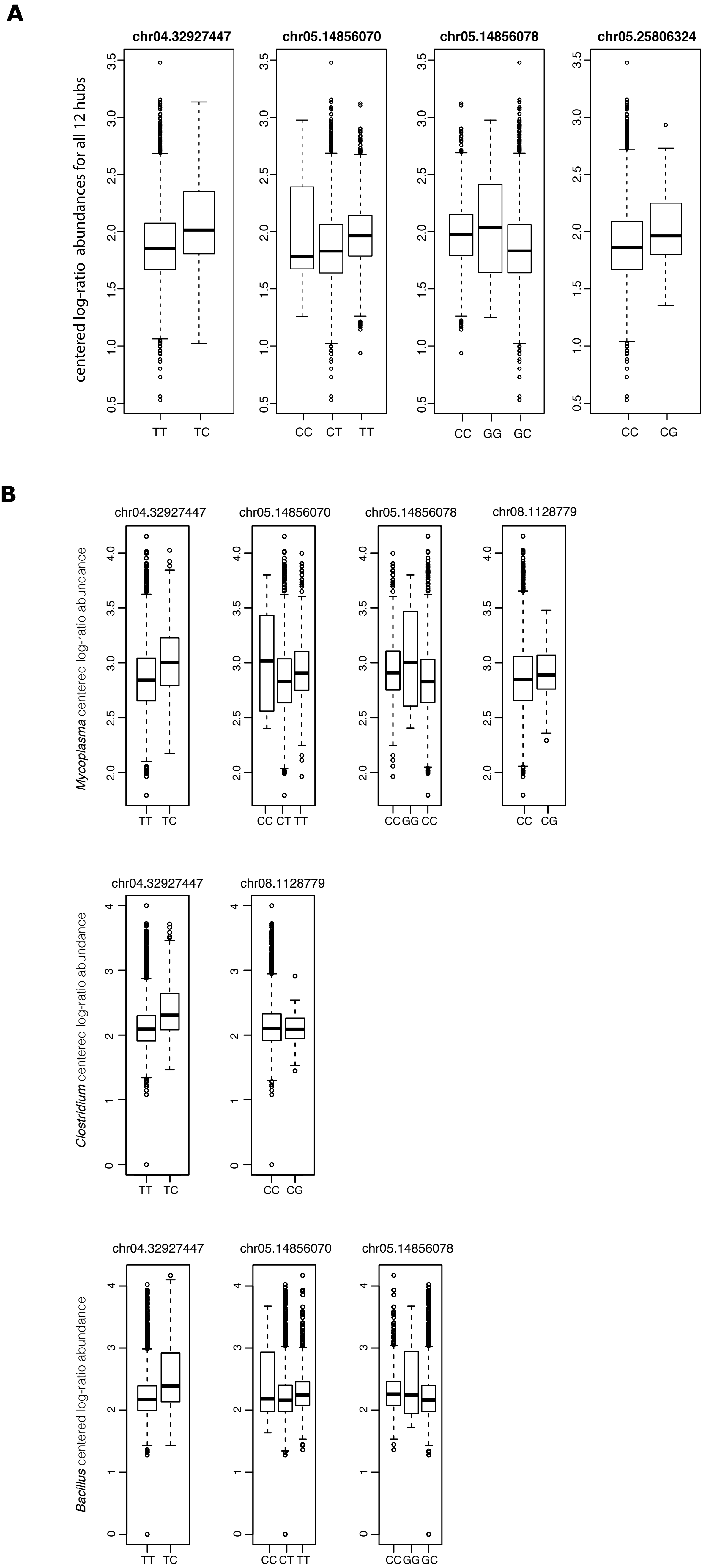

Figure S4. Relationships between significant SNPs and hubs abundances represented as box plots.

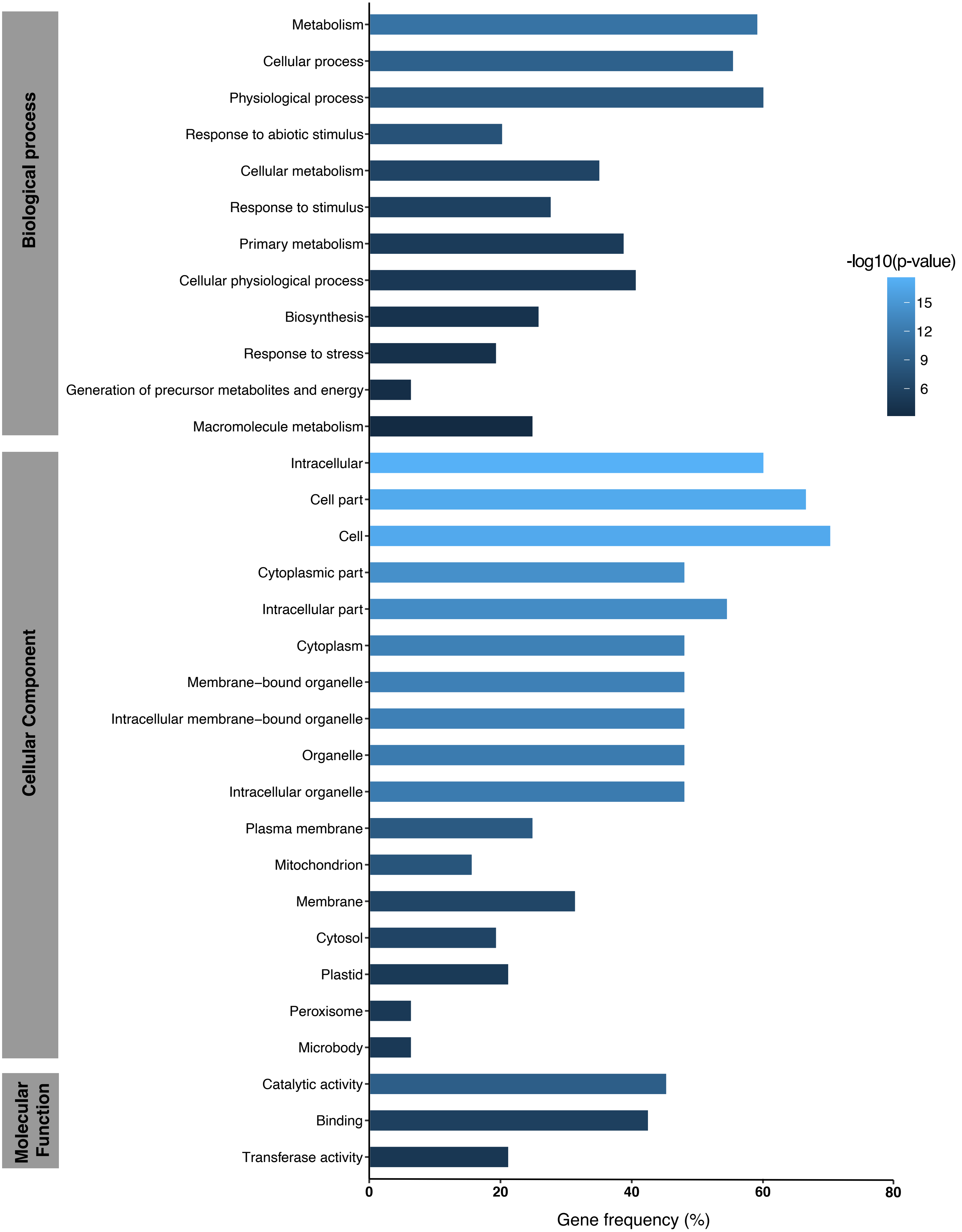

Figure S5. Gene ontology enrichment analysis with all genes from the hapoblocks.
