# Supplementary material for: Characterization of the Leaf Microbiome from Whole-Genome Sequencing Data of the 3000 Rice Genomes Project
Source: Rice (N Y). 2020 Oct 9;13:72. doi: 10.1186/s12284-020-00432-1 (PMC7547056; doi:10.1186/s12284-020-00432-1)
Supplement: Supplementary file 2 — Additional file 2. Methods. [file 12284_2020_432_MOESM2_ESM.docx]

**Characterization of the leaf microbiome from whole-genome sequencing data of the 3000 rice genomes project.**

Veronica Roman-Reyna^acd*^, Dale Pinili^ad^, Frances Nikki Borja^a^, Ian Lorenzo Quibod^a^, Simon C. Groen^b^, Nikolai Alexandrov^a^, Ramil Mauleon^a^, Ricardo Oliva^a*^.

**Materials and Methods**

**Genomic source**

To describe the rice leaf microbiome, we used the 3,000 Rice Genomes Project database (The 3,000 rice genomes project 2014) (Additional file 1: Figure S1). This database was originally created to gather information about rice genetic variation. We used the DNA raw reads from 3,024 germplasm sequenced accessions to gather information about the rice leaf microbiome (The 3,000 rice genomes project 2014). We used the reads that did not mapped to rice genomes as potential microbial reads (Gouin et al. 2015; Kamitani et al. 2016). We first mapped the raw leaf genome sequences of each accession to the five rice reference genomes (Nipponbare, 93-11, IR64, Kasalath, and DJ123) with the software BWA v0.7.10 (Li and Durbin 2009). Then, we extracted the reads that did not map to the five rice genomes with samtools v1.0 (Li et al. 2009). Finally, the unmapped reads were converted to Fasta files with BEDtools v2.17.0 (Quinlan and Hall 2010) and were used as the rice leaf microbiome. To assign taxonomy labels to the unmapped reads we used the software Kraken v1.0 (Wood and Salzberg 2014). This software classified the reads, using exact k-mer matches to a trained database. The database included the Bacteria and Archaea genomes from RefSeq NCBI database (release 69). To estimate taxa abundance we used the Bayesian-based tool Bracken v1.0 (Lu et al. 2017). To reduce the rarest species that might contribute to noises in further comparisons, we used the same cutoff as Wagner et al (2016); we kept the genera that were present in at least 10% of samples across all locations. We used this cutoff for diversity estimations, functional analysis and Genome Wide Association Studies.

**Diversity estimation**

To compare the microbial composition across all rice accessions, we used the relative abundance normalization on the count matrix, where the read counts for a taxa-level in a given sample were divided by the sum of all counts in that sample. This approach took into account the differences in read depth for each accession. The count matrix of the unmapped reads was generated by the tool Bracken v1.0 (Lu et al. 2017). We did not use other normalization approaches like variance-stabilizing transformation, rarefaction or centered log-ratio transformation because our sample size was high. To calculate the richness and diversity indices in all rice leaf accessions, we used the Vegan v2.5-3 R package. To identify the taxa that contribute to dissimilarities between environments, we used the *simper* function from the Vegan v2.5-3 R package. We used the relative abundances of Genera in all accessions as *community data matrix*, the environment as the *group structure* and we set to 100 *permutations*. Based on the matrix the *simper* function calculated the contributions of each taxon to the overall Bray-Curtis dissimilarity and the output was a list of cumulative contributions ordered by taxa.

**Quantification of 16S from abundant genera**

To validate the results from 3K-RGP metagenome analysis, we amplified and quantified 11 of the most abundant genera in 17 rice accessions from the 3K-RGP. To select the accessions, we used the accessions grew in the Philippines. Then we performed an abundance cluster analysis for each rice variety and select accessions that represent each cluster. Therefore, for Indica we selected five accessions that belong to different clusters. With the same criteria we selected four accessions for Japonica, two accessions for Aus, four accessions for Admix, and two for Aromatic. These 17 accessions have different microbiome profiles, allowing us to test the 11 abundant genera. For this validation, we sown 12 seeds per accession in glasshouse conditions at The International Rice Research Institute and harvested 21 days old leaves. We pooled four leaves from four plants as one biological replicates. We had three biological replicates. We cleaned the leaves with 70 % ethanol, 2 % bleach, and water before DNA extraction. DNA was extracted with CTAB method (The 3,000 rice genomes project 2014). The DNA was aliquoted in similar concentrations (20 ng/uL) for the qPCR. For amplification and quantification, we used the StepOnePlus™ Real-Time PCR System and SYBR^(R)^ Green I dye following manufacturer protocol (Applied Biosystems, USA). Briefly, we used a final reaction volume of 10 uL with a primer final concentration of 200 nM. We ran three biological and three technical replicates. We used the default parameters for a comparative C_T_ method setup, with maximum 40 cycles. We selected published primers for *Pseudomonas* sp., *Burkholderia* sp., *Mycoplasma* sp*., Streptomyces* sp., *Methylobacterium* sp. We designed primers for *Mycobacterium* sp., *Xanthomonas sp., Alteromonas sp., Pantoea sp., Bacillus sp. and Clostridium sp*. (Additional file 3: Table S3). To design the primers, we created a consensus alignment with the available 16S rDNA sequences at NCBI for a specific bacterial genus using MEGA7 (Kumar et al. 2016). We used the consensus sequence to design primers using the Primer-BLAST tool from NCBI (Ye et al. 2012). For comparisons, all samples were normalized to the 16S rDNA region V3-V4.

**Microbial ecological network and functional analysis**

To infer graphical models for the rice microbial ecological network, we used SpiecEasi v0.1.4 R package (Kurtz et al. 2015). We used the absolute counts, as the program does a centered log-ratio transformation. As the program was sensitive to low abundant and low incidence taxa, we tested the effect of removing the genera not present in at least 10%, 30% and 50% of all the samples from the count matrix. We compared the network properties in the different percentages, and we decided to keep taxa present in at least 50% of all samples based on the network stability scores, that were higher in 50 % compared to 10% and 30%. We did the analysis with the Meinshausen-Buhlmann's neighborhood method and the following parameters, lambda.min.ratio=1e-2, nlambda=30, pulsar.params=list (rep.num=100, ncores=7) (Kurtz et al. 2015). The network was plotted with the software Gephi v0.9.2. To predict the functional profile we used the web-based tool Vikodak (now ivikodak, <https://web.rniapps.net/iVikodak/index.php>). Briefly, this algorithm is based on the assumption that genes present in various microbes contributed to a specific pathway(s) in the microbial community. under the co-metabolism algorithm workflow (Nagpal et al. 2016). We used this approach because microbial community functions are dependent on bacterial richness and interaction among microbes. For this analysis we focused on Bacterial functions, therefore, to remove pathways originating from eukaryotic sources we implemented a Pathway Exclusion Cut-off. We used the modules Global Mapper, and ‘Co-metabolism’ algorithm. The pathways were classified with KEGG hierarchy levels.

**Genome-wide Association Study**

To identify rice genetic factors that determine the compositions of microbial communities, we performed a Genome-wide Association Study (GWAS). We first determined the rice population stratification using the software PLINK v1.9. With the software GEMMA v0.9 we determine the kinship matrix and then we ran a SNP-based association test (Purcell et al. 2007; Zhou and Stephens 2012). To avoid association bias due to lack of information for most of Chinese accessions (agCh), we kept agCh and agPh together for the analysis. We used 6.5 million filtered rice SNPs from the 29 million bi-allelic SNPs retrieved from the Rice SNP-Seek (3kRG full (29mio) biallelic SNP set v.4, snp-seek.irri.org). We excluded SNPs with the lower genotypic rate (> 95%) and minor allele frequency (MAF < 0.01). We removed the SNPs that fail the Hardy-Weinberg equilibrium test (P < 0.0001). We performed a simultaneous GWAS with the abundance of the hubs: *Clostridium, Mycoplasma, Bacillus, Buchnera, Prochlorococcus, Helicobacter, Methylobacterium, Chamaesiphon, Azotobacter, Kineococcus, Acidovorax, and Pseudomonas.*

For GWAS, we used the centered log-ratio (clr) transformation of hub abundances to keep the same approach used for building ecological networks. We ran GWAS with the GEMMA multivariable linear model (mlm) and identified significant SNPs by filtering with False Discovery Rate (FDR <0.01) and P-value (P-value <1E-15). GEMMA mlm allows using multiple phenotypic traits at the same time, therefore we used the abundance of the 12 hubs. For the association analysis, we estimated the relatedness matrix using the PLINK binary ped and then performed an eigen-decomposition. The Manhattan plots and quantile-quantile (Q-Q) plot were created with qqman v.0.1.3 R package. We determined the expected and observed probabilities of SNPs association with Q-Qplot. We grouped the significant SNP signals by haploblocks based on the linkage disequilibrium decay (LD<0.3) and correlation coefficients (*r^2^* > 0.6) in each chromosome using Haploview v4.2 (Gabriel et al. 2002; Barrett et al. 2005). We identified and described the genes by gene ontology annotation, QTL overlapping, and SNP effect based on the information from SNP-Seek Database (snp-seek.irri.org). To do the gene ontology (GO) enrichment analysis we used the RiceNetDB database (Liu et al. 2013). We kept GO categories based on p-value (<0.01). False discovery rate (FDR = 0) and false positive (fp= 0). The relationship between significant SNPs and hubs abundance was visualized by boxplots and validated by paired-wilcoxon analysis (p-value < 0.05). We obtained the haplotypes for all 3024 rice accessions from the website snp-seek.irri.org.

**References**

Barrett JC, Fry B, Maller J, Daly MJ (2005) Haploview: analysis and visualization of LD and haplotype maps. Bioinformatics 21:263–265. https://doi.org/10.1093/bioinformatics/bth457

Gabriel SB, Schaffner SF, Nguyen H, et al (2002) The structure of haplotype blocks in the human genome. Science 296:2225–2229. https://doi.org/10.1126/science.1069424

Gouin A, Legeai F, Nouhaud P, et al (2015) Whole-genome re-sequencing of non-model organisms: lessons from unmapped reads. Heredity 114:494–501. https://doi.org/10.1038/hdy.2014.85

Kamitani M, Nagano AJ, Honjo MN, Kudoh H (2016) RNA-Seq reveals virus–virus and virus–plant interactions in nature. FEMS Microbiol Ecol 92:. https://doi.org/10.1093/femsec/fiw176

Kumar S, Stecher G, Tamura K (2016) MEGA7: Molecular Evolutionary Genetics Analysis version 7.0 for bigger datasets. Mol Biol Evol msw054. https://doi.org/10.1093/molbev/msw054

Kurtz ZD, Müller CL, Miraldi ER, et al (2015) Sparse and compositionally robust inference of microbial ecological networks. PLOS Comput Biol 11:e1004226. https://doi.org/10.1371/journal.pcbi.1004226

Li H, Durbin R (2009) Fast and accurate short read alignment with Burrows-Wheeler transform. Bioinforma Oxf Engl 25:1754–1760. https://doi.org/10.1093/bioinformatics/btp324

Li H, Handsaker B, Wysoker A, et al (2009) The Sequence Alignment/Map format and SAMtools. Bioinformatics 25:2078–2079. https://doi.org/10.1093/bioinformatics/btp352

Liu L, Mei Q, Yu Z, et al (2013) An Integrative Bioinformatics Framework for Genome-scale Multiple Level Network Reconstruction of Rice. J Integr Bioinforma 10:94–102. https://doi.org/10.1515/jib-2013-223

Lu J, Breitwieser FP, Thielen P, Salzberg SL (2017) Bracken: estimating species abundance in metagenomics data. PeerJ Comput Sci 3:e104. https://doi.org/10.7717/peerj-cs.104

Nagpal S, Haque MM, Mande SS (2016) Vikodak - A modular framework for inferring functional potential of microbial communities from 16S metagenomic datasets. PLOS ONE 11:e0148347. https://doi.org/10.1371/journal.pone.0148347

Purcell S, Neale B, Todd-Brown K, et al (2007) PLINK: A tool set for whole-genome association and population-based linkage analyses. Am J Hum Genet 81:559–575

Quinlan AR, Hall IM (2010) BEDTools: a flexible suite of utilities for comparing genomic features. Bioinformatics 26:841–842. https://doi.org/10.1093/bioinformatics/btq033

The 3,000 rice genomes project (2014) The 3,000 rice genomes project. GigaScience 3:7. https://doi.org/10.1186/2047-217X-3-7

Wood DE, Salzberg SL (2014) Kraken: ultrafast metagenomic sequence classification using exact alignments. Genome Biol 15:R46. https://doi.org/10.1186/gb-2014-15-3-r46

Ye J, Coulouris G, Zaretskaya I, et al (2012) Primer-BLAST: A tool to design target-specific primers for polymerase chain reaction. BMC Bioinformatics 13:134. https://doi.org/10.1186/1471-2105-13-134

Zhou X, Stephens M (2012) Genome-wide efficient mixed model analysis for association studies. Nat Genet 44:821–824. https://doi.org/10.1038/ng.2310
